# Non-muscle myosin II is required for correct fate specification in the *Caenorhabditis elegans* seam cell divisions

**DOI:** 10.1038/s41598-017-01675-7

**Published:** 2017-06-14

**Authors:** Siyu Serena Ding, Alison Woollard

**Affiliations:** 1 Department of Biochemistry, University of Oxford, South Parks Road, Oxford, OX1 3QU United Kingdom; 2 Institution of Clinical Sciences (ICS), Faculty of Medicine, Imperial College London, Du Cane Road, London, W12 0NN United Kingdom; 3 MRC London Institute of Medical Sciences (LMS), Hammersmith Hospital Campus, Du Cane Road, London, W12 0NN United Kingdom

## Abstract

During development, cell division often generates two daughters with different developmental fates. Distinct daughter identities can result from the physical polarity and size asymmetry itself, as well as the subsequent activation of distinct fate programmes in each daughter. Asymmetric divisions are a feature of the *C. elegans* seam lineage, in which a series of post-embryonic, stem-like asymmetric divisions give rise to an anterior daughter that differentiates and a posterior daughter that continues to divide. Here we have investigated the role of non-muscle myosin II (*nmy-*2) in these asymmetric divisions. We show that *nmy-2* does not appear to be involved in generating physical division asymmetry, but nonetheless is important for specifying differential cell fate. While cell polarity appears normal, and chromosome and furrow positioning remains unchanged when *nmy-2* is inactivated, seam cell loss occurs through inappropriate terminal differentiation of posterior daughters. This reveals a role for *nmy-2* in cell fate determination not obviously linked to the primary polarity determination mechanisms it has been previously associated with.

## Introduction

Asymmetric cell division generates two daughters that adopt distinct fates, a fundamental process in developmental biology that allows a single fertilised cell to give rise to a multi-cellular organism with diverse cell types^1–3^. The term “asymmetrical cell division” itself encompasses multiple levels of asymmetry, including physical size asymmetry as well as downstream differential fate specification. These two aspects of the division are linked yet somewhat separable. Physical division asymmetry concerns processes such as polarity establishment prior to division, spindle positioning and division site specification, in order to ensure the correct partitioning of the genetic material and cytoplasmic contents. Differential fate specification, on the other hand, may be influenced by the localisation and function of intrinsic fate determinants, or by signalling from the surrounding environment. Cooperation between physical size asymmetry and differential fate specification together ensures the correct outcome of asymmetric divisions.

**Figure 1:**
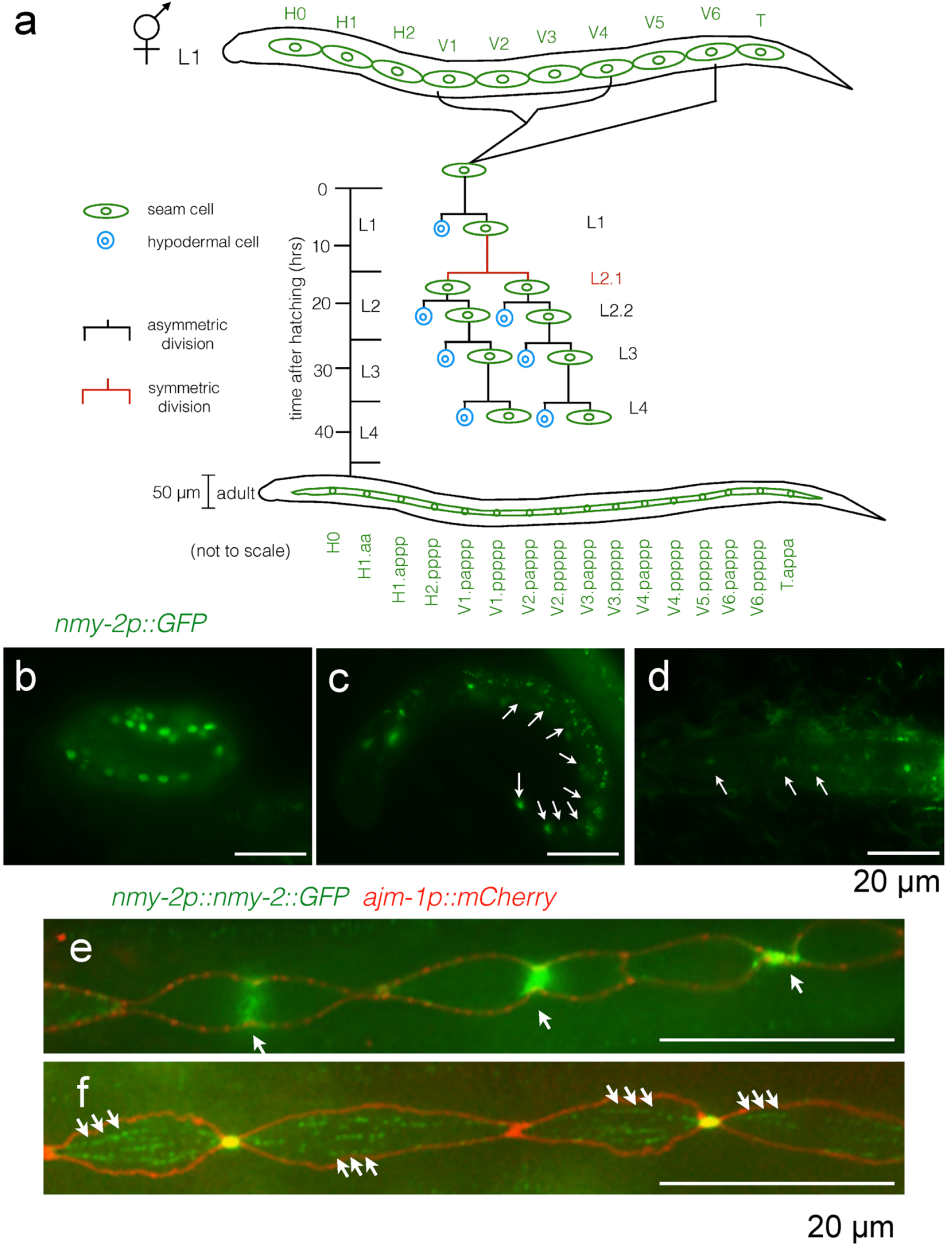
*nmy-2* is expressed in the seam. (**a**) Hermaphrodite lineage diagram of the V seam cells (V1-4, V6). Seam cells are green and differentiated hypodermal cells are blue. The worms hatch with ten seam cells on each side. The V lineage cells undergo L1, L2.2, L3 and L4 asymmetric divisions (white lines) as well as the L2.1 symmetric division (brown lines) during larval development to generate a total of 16 terminal seam cells per side, which fuse to form a seam syncytium in adulthood, whilst contributing hypodermal cells to the hyp7 syncytium to allow the worm to grow in size. The adult worm is not drawn to scale compared to the size of the L1 larva. (**b**–**d**) The *nmy-2* transcriptional reporter (strain AW1118) shows seam-specific expression in the (**b**) three-fold embryo, (**c**) L1 larva, and (**d**) L2 larva. Arrows point to seam cells. (**e**,**f**) The *nmy-2* translational reporter with an integrated apical junction marker (*ouIs21*[*ajm-1p::mCherry*]) (strain AW1092) shows that NMY-2 localises to the cytokinesis furrow (arrows in **e**) and to punctae forming fibre-like structures (arrows in (**f**). (**e**) Shows a larva undergoing the L3 division, and (**f**) is a larva that had just completed the L4 division. Larvae performing the L2.2 asymmetric division showed similar expression patterns and are omitted for simplicity. As for all the microscopy images here and henceforth, anterior is to the left, posterior is to the right, dorsal is on the top, and ventral is on the bottom.

*Caenorhabditis elegans* seam cells provide a valuable system to study the regulation of asymmetric divisions in a stem-like lineage. Seam cells consist of two lateral rows of multipotent, neuroectodermal cells that lie along the length of the animal. Worms hatch with ten seam cells on each side (H0, H1-2, V1-6, and T) (see Fig. 1a for the lineage diagram). During hermaphrodite larval development, V lineage seam cells divide once asymmetrically during each of the four larval stages, typically generating a differentiated anterior daughter that rounds up and moves out of the seam line, fusing with the hyp7 syncytium, and a posterior seam daughter that retains its characteristic eye shape and subsequent proliferative capability. Additionally, at the beginning of the second larval stage, V lineage seam cells undergo a single symmetric division (L2.1 division) before the L2 asymmetric division (L2.2 division). This symmetric division generates two proliferative seam daughters, thereby expanding the number of seam cells to 16^4^. At the L4 to adulthood transition, the 16 seam cells on each side of the worm terminally differentiate by undergoing homotypic fusion to form a seam syncytium^5^ (Fig. 1a). During adulthood, the seam syncytium secretes alae, a set of raised longitudinal ridges on the exterior of the worm, whose presence correlates with correct terminal differentiation of the seam cells^6–8^.

Several pathways and factors are known to regulate different aspects of post-embryonic seam development. The Wnt/β-catenin asymmetry pathway determines the polarity of most somatic divisions occurring along the anterior-posterior (A-P) axis in *C. elegans* ^9^, and seam cells are no exception^10^. In brief, seam cells become polarised at division when WRM-1 (β-catenin) becomes enriched at the anterior cortex due to increased microtubule trafficking from the nucleus^11^. In the posterior nucleus, WRM-1 levels remain high, causing the Wnt effector POP-1 (TCF/LEF-1) to be exported from the nucleus^12, 13^, thus lowering the ratio of POP-1 to SYS-1 (a second *C. elegans* β-catenin and POP-1 co-activator) in the nucleus of posterior daughters^14^. This low POP-1: high SYS-1 ratio activates POP-1 to upregulate Wnt target genes in posterior daughters to specify the proliferative fate^14–16^. In contrast, anterior daughters, with their high POP-1: low SYS-1 ratio (maintained by low levels of nuclear WRM-1 failing to trigger POP-1 export) repress Wnt targets and thus differentiate. Disruption of Wnt pathway components leads to abnormal seam cell numbers. Inactivating *wrm-1* reduces adult seam cell number to as low as three per side since posterior seam daughters mimic the anterior fate and differentiate inappropriately, whereas *pop-1* silencing generates up to 67 seam nuclei per side by transformation of anterior daughters to adopt the posterior, proliferative fate^10^. Although Wnt pathway component asymmetry is required for asymmetric seam cell divisions, it can be by-passed. For example, during the L2.1 symmetrical seam cell division in which both daughters adopt the proliferative fate, POP-1 and WRM-1 asymmetry are still observed^17, 18^.

While Wnt signalling primarily concerns cell polarity establishment acting upstream of differential fate specification, several transcription factors, including RNT-1 and BRO-1 as well as CEH-20 and UNC-62, are thought to regulate seam cell fate patterning independently of Wnt signalling. For example, RNT-1 and its binding partner BRO-1 (the sole *C. elegans* homologues of RUNX and CBFβ, respectively) act to promote the proliferative fate in posterior seam daughters by inhibiting CKI-1, a cyclin E/CDK-2 inhibitor^19–22^. Thus *rnt-1* and *bro-1* mutants both have fewer seam cells due to failed proliferation, but inactivating *rnt-1* or *bro-1* does not suppress the seam cell hyperplasia associated with *pop-1* knock down^10^, suggesting RNT-1/BRO-1 are unlikely to be Wnt targets. Mutants of *ceh-20* and *unc-62* (Pbx and Meis TALE-class transcription factors, respectively, which also form a transcripitonal partnership regulating seam lineage divisions) have the opposite phenotype of *rnt-1* and *bro-1* mutants, displaying seam cell hyperplasia. This is not supressed in a *wrm-1* temperature sensitive (ts) mutant background^18^, again suggesting that *ceh-20*/*unc-62* likely function in a separate pathway to Wnt. However, the hyperplasia caused by *ceh-20*/*unc-*62 perturbation is completely suppressed when *rnt-1* and/or *bro-1* are inactivated, suggesting that *ceh-20*/*unc-62* likely function upstream of *rnt-1*/*bro-1* in a common pathway of seam cell fate patterning, with CEH-20/UNC-62 repressing RNT-1 in the anterior daughter to promote differentiation rather than proliferation^18^.

Non-muscle myosin II (NMY-2) is a conserved motor protein that bundles actin to provide the contractile forces needed for a wide range of biological processes, including cell migration and adhesion^23^, as well as cytokinesis^24–26^. It has emerged as a key regulator of *C. elegans* asymmetric divisions in three contexts. First, in the *C. elegans* zygote, *nmy-2* is required to establish and maintain cell polarity, its protein product becoming anteriorly localised following sperm entry to direct localised actomyosin contraction to transport PAR-3, PAR-6 and PKC-3 to the anterior^25, 27–29^. PAR proteins establish exclusive anterior and posterior domains by mutual inhibition, and *nmy-2* is required throughout the cell cycle to maintain PAR polarity^30^. Later on in the four-cell stage embryo, *nmy-2* is required for endoderm specification in the EMS division. The P2 and EMS sisters signal to each other to align the EMS division orientation along the A-P axis and induce asymmetric division^31^. P2/EMS signalling uses inputs from both the Wnt and the Src pathways^32^, and *nmy-2* has been shown to promote Src-dependent phosphotyrosine signalling to specify endoderm from the EMS division in parallel to Wnt signaling^30^. Beyond embryogenesis, a post-embryonic role of *nmy-2* was demonstrated in the asymmetric division of the Q.a neuroblast. NMY-2 preferentially localises to the anterior cortex during anaphase, ‘squeezing’ the anterior half of the cell and allowing the posterior to expand like a balloon in response. As a result, the division produces a smaller anterior daughter that undergoes apoptosis and a larger posterior daughter that survives^33^. *Drosophila melanogaster* neuroblasts utilise a strikingly similar mechanism in order to generate a larger apical daughter and a smaller basal daughter using polarised cortical myosin II^34, 35^. Given the diverse roles of *nmy-2* in asymmetric cell divisions, we asked whether *nmy-2* also functions in the *C. elegans* seam, a unique case of a post-embryonic stem-like lineage that sets it apart from the other *C. elegans* lineages described above.

## Results

### *nmy-2* is expressed in the seam

In order to examine whether *nmy-2* is likely to play a role in seam cell development, we first confirmed its expression in the seam. The transcriptional reporter (*nmy-2p::gfp*) showed strong expression in seam cells in the three-fold embryo as well as in L1 and L2 larvae (Fig. 1b–d, arrows). *nmy-2* expression in the late-stage embryo is consistent with the NMY-2 regulatory light chain localisation in a previous report^36^ and its known role in seam-dependent embryonic elongation^36–38^. Seam-specific expression seemed to decrease from embryo to larvae, becoming obscured by strong expression in other tissues during adulthood, including head neurons, the vulva, the posterior intestine, and the rectal gland (Supplementary Fig. S1).

The translational reporter (*nmy-2p::nmy-2*::*gfp*; a kind gift of Dan Dickinson, University of North Carolina, Chapel Hill, USA) generated using CRISPR-mediated GFP knock-in ref. 39 was used to visualise endogenous NMY-2 protein, with the integrated apical junction marker (*ajm-1p::mCherry*) crossed into the strain to outline the seam cells. NMY-2 was observed to localise to the ingressing cleavage furrow (Fig. 1e, arrows) as expected given its role in cytokinesis. Additionally, NMY-2 punctae were observed in an aligned fibre-like conformation along the A-P axis of cells (Fig. 1f, arrows). These fibres are likely cytoplasmic because they did not co-localise with the apical junction marker outlining the lateral cell cortex (in red), although we cannot exclude the possibility that the fibres in fact covered the apical cortex of seam cells.

Taken together, the transcriptional and translational reporters confirmed *nmy-2* expression in the seam that is consistent with its previously reported functions in embryonic elongation and cytokinesis.

### *nmy-2* knockdown causes progressive seam cell loss following asymmetric divisions

**Figure 2:**
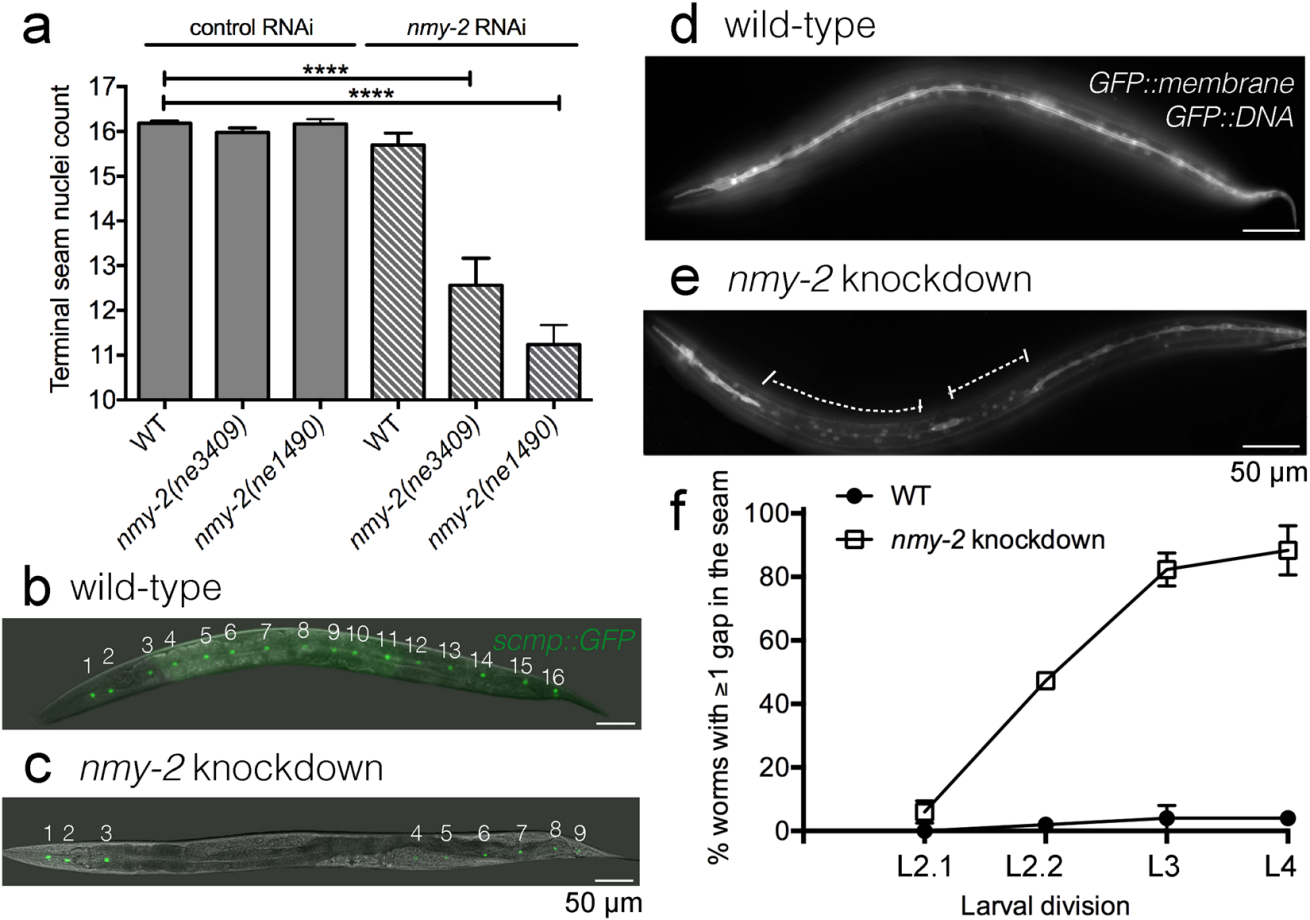
Post-embryonic *nmy-2* knockdown results in progressive seam cell loss following asymmetric divisions. (**a**) Combining the ts allele with RNAi was necessary to achieve a decrease of terminal seam nuclei numbers. Synchronised L1 larvae (hatched from animals grown at 15 °C) with or without the *nmy-2*(*ne3409*) *or nmy-2*(*ne1490*) ts mutations (strains AW335, AW785 and AW786, respectively) were fed control or *nmy-2* RNAi at 25 °C. Seam nuclei were counted in young adults using the integrated seam cell marker *wIs51*[*scmp::gfp*]. The *y*-axis starts at 10 because the L1 larvae are born with 10 seam cells and gain additional seam cells through post-embryonic development. “WT” = wild-type. n > 50 for each sample. Error bars represent the standard error of the mean (SEM). Unpaired, two-tailed *t*-test was used to determine significance (****p < 0.0001). At least three independent replicates of the experiment were performed. Hashed pattern here and henceforth represents *nmy-2* RNAi treatment. (**b**,**c**) Wild-type and *nmy-2* knockdown adult hermaphrodites with the *wIs51* seam cell marker. The wild-type animal showed 16 seam nuclei on one side, and the *nmy-2* knockdown animal had 9. (**d**,**e**) Wild-type and *nmy-2* knockdown adult hermaphrodites with the integrated membrane and DNA markers *heIs63*[*wrt-2p::gfp::PH;wrt-2p::gfp::H2B*]. The wild-type animal showed a smooth and continuous seam syncytium, and the *nmy-2* knockdown animal showed two gaps in the seam syncytium (dashed lines). (**f**) The percentage of worms with at least one gap in the seam was quantified for the control and *nmy-2* knockdown conditions following each seam cell division from L2 onwards (strains SV1009 and AW788). L2.1 = L2 first (symmetric) division. L2.2 = L2 second (asymmetric) division. n > 50 per strain per stage. Two independent replicates of the experiment were performed.

Having confirmed the expression of *nmy-2* in the seam cells, we next investigated the effect of inactivating *nmy-2* on seam cell development during the larval stages. Two temperature-sensitive alleles *nmy-2*(*ne3409ts*) and *nmy-2*(*ne1490ts*) produced no obvious phenotype at 15 °C but were 100% embryonic lethal at the restrictive temperature of 25 °C (n > 200). Shifting L1 larvae to the restrictive temperature, however, produced no effect on the terminal seam nuclei count, as assayed using the integrated seam cell marker *scmp::gfp* (Fig. 2a, p = 0.06 for *ne3409* and p = 0.39 for *ne1490*). Given that both loss-of-function ts alleles impair the S2 region of the myosin molecule^30^, it is not surprising that the results are similar between the two. Since S2 is required for dimerisation and head-head coupling^40^, NMY-2 motor activity appears dispensable for post-embryonic seam cell divisions. A second tool for knocking down *nmy-2* is RNA interference (RNAi). While RNAi of L4 worms produced high embryonic lethality as well as a significant reduction of seam nuclei count in surviving progeny (Supplementary Fig. S2), post-embryonic treatment again produced little effect on the seam (Fig. 2a, p = 0.07). Combining either ts mutation with post-embryonic RNAi treatment, however, produced a robust and significant reduction of terminal seam cell number from 16.2 per side in control animals (Fig. 2b) to 11.2–12.5 per side in treated animals (Fig. 2a, p < 0.0001; Fig. 2c). Seam cells were observed to be absent anywhere along the length of the treated worm. This combined ts and RNAi treatment regime was used to achieve post-embryonic *nmy-2* knockdown for all subsequent experiments, with *nmy-2*(*ne3409*) as the allele of choice because it is the reference allele previously used in a number of embryonic studies.

Consequent to having fewer seam cells, *nmy-2* knockdown led to gaps in the seam, which were easily visualised using the integrated membrane and DNA markers (*wrt-2p::gfp::PH; wrt-2p::gfp::H2B*, driven by the highly seam-specific *wrt-2* promoter). While the wild-type animals had a smoothly fused seam syncytium in adulthood (Fig. 2d), *nmy-2* knockdown animals often had clear gaps in the seam (Fig. 2e, dashed lines), presumably a result of seam cell loss and the inability of the remaining seam cells to re-establish cell-cell contact. Variable numbers and sizes of gaps were seen along the length of the worm. Quantification of gaps after each seam cell division revealed that while gaps were rare following the L2.1 symmetric division, they became progressively more common after each asymmetric division (L2.2, L3, L4) (Fig. 2f). Quantification was not performed after the L1 asymmetric division, because the elapsed feeding time was likely insufficient for producing an RNAi effect. After the L4 division, 88% of the *nmy-2* knockdown worms had at least one gap in the seam syncytium, indicating that seam cell loss is a highly penetrant phenotype amongst individual animals. Using the same set of images, the effect of *nmy-2* inactivation is also reported as the number of seam nuclei following each division (Supplementary Fig. S3), which illustrates continuous seam cell loss following each asymmetric division. The plateau seen after the L4 division in Fig. 2f is therefore an artefact of reporting the penetrance as per animal, since multiple seam cells may be lost from a single animal. When considered at the level of penetrance per cell division, 0.9, 2.0, and 2.3 seam cells were lost following the L2.2, L3, and L3 division, respectively, accounting for a phenotypic penetrance of 5.6%, 13.1%, and 17.3% per cell division, respectively (Supplementary Fig. S3).

### *nmy-2* knockdown may not affect physical division asymmetry

Does *nmy-2* perturbation cause seam cell losses by affecting size asymmetry during division? To examine this possibility, we measured the positioning of the chromosomes (labelled using the *wrt-2::gfp::H2B* DNA marker) with respect to the ends of the cell and the ingressing furrow (labelled using the *ajm-1p::mCherry* apical junction marker) as a proxy for both mitotic spindle and cleavage furrow positioning. The measurements were taken at interphase, prophase, metaphase, and anaphase of the L2.1 symmetric, L2.2 asymmetric, and L3 asymmetric divisions for both the wild-type (Supplementary Fig. S4) and the *nmy-2* knockdown (Supplementary Fig. S5) scenarios (see Supplementary Methods for more details on the experimental rationale and methodology). The *nmy-2* knockdown chromosomal positioning dataset was very similar to the wild-type data in terms of nuclear and spindle positioning and cleavage site location, suggesting that *nmy-2* may not be involved in generating size asymmetry during seam cell divisions. In addition, the division orientation was normal along the A-P axis despite *nmy-2* inactivation. It is conceivable, however, that the lack of overt defect in most cells may be due to the relatively low penetrance of the seam cell loss per cell division (Supplementary Fig. S3). Thus we cannot completely rule out the possibility that *nmy-2* has a role in physical division asymmetry.

Nevertheless, these previously unreported chromosomal positioning data revealed several intriguing properties of normal seam cell biology observed in wild-type animals, as discussed in Supplementary data (Fig. S4 legend). In particular, it is worth noting that spindle displacement does not appear to be the main mechanism for generating different daughter sizes in seam cell divisions. For example, in the L3 division, the spindle complex as a whole is slightly displaced towards the posterior in Vn.pap (p = 0.04) and towards the anterior in Vn.ppp (p = 10^−5^) whilst both cytokinesis furrows are perfectly centred (p = 0.42 for Vn.pap and p = 0.34 for Vn.ppp) (Supplementary Fig. S4). The slight difference this generates in the nascent daughters (relatively sizes of 52, 48, 47 and 55 arbitrary units for Vn.papa, Vn.papp, Vn.pppa, Vn.pppp, respectively) immediately following the division is unlikely to sufficiently account for the obvious size difference later on (Supplementary Fig. S6). Thus the re-sizing and re-shaping of seam division daughters appear to take place primarily post-division rather than during division via spindle displacement, and in any case appears to be unaffected by abrogating *nmy-2*.

### Seam cell loss in *nmy-2* knockdown is due to cell fate mis-specification

**Figure 3:**
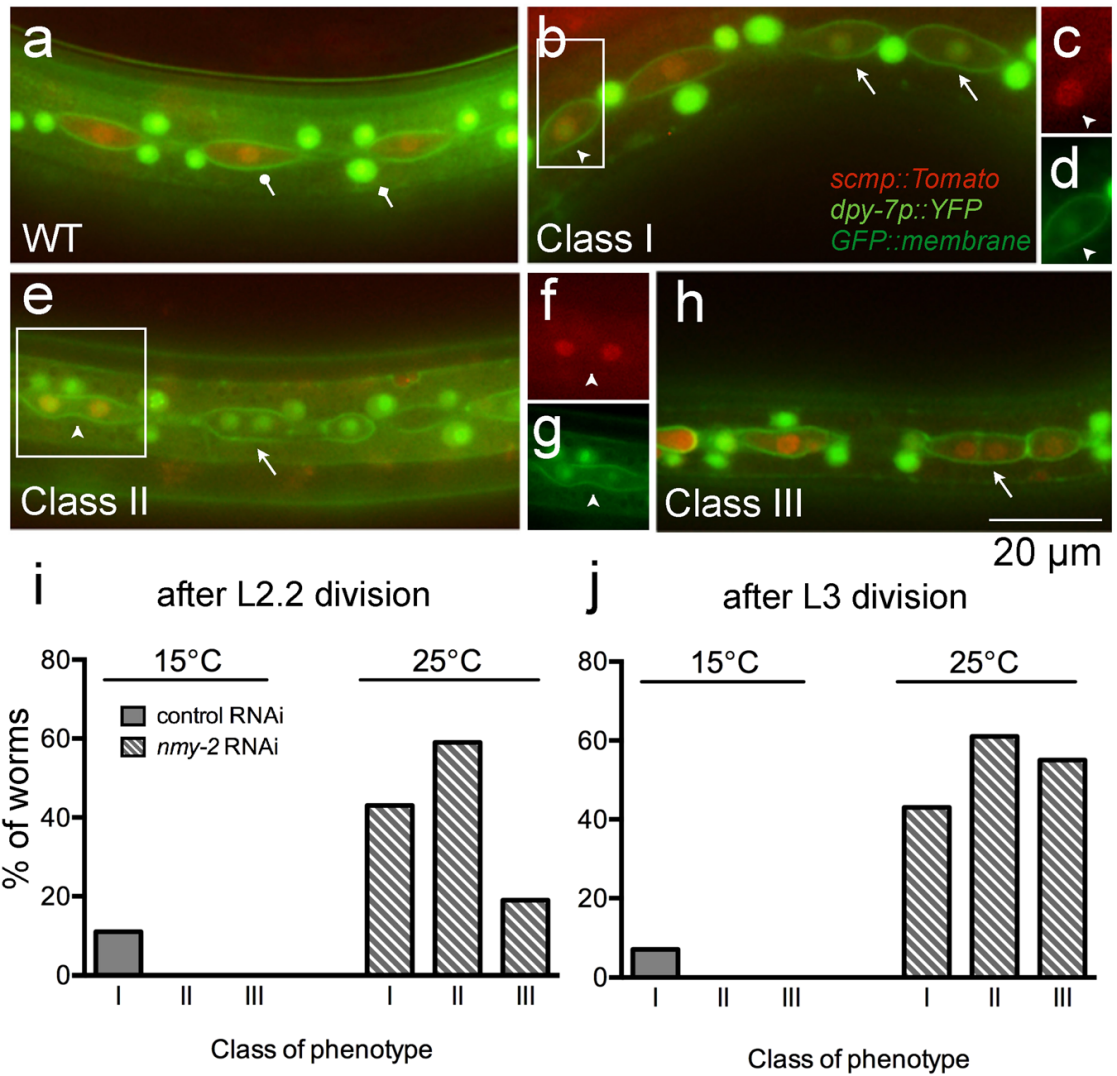
*nmy-2* knockdown produces three distinct classes of cell fate mis-specification phenotypes. (**a**) The dual-colour fate reporter strain (AW1015) contains *ouIs10*[*scmp::NLS::tdTomato; dpy-7p*::*2xNLS*::*YFP*;*wrt-2p::GFP*::*PH*] that marks the seam nuclei red (lollipop arrow) and the hypodermal nuclei green (diamond arrow). (**b**–**h**) *nmy-2* knockdown produced distinct classes of fate transformation phenotypes (arrows and arrowheads) (strain AW1018). The animals shown here were imaged after the L2.2 asymmetric division. Arrows point to pertinent cells representing each Class; arrowheads point to cells clearly expressing both fate markers. (**c**) and (**d**) Are red and green channel images corresponding to the boxed area in (**b**), respectively; (**f**) and (**g**) are red and green channel images corresponding to the boxed area in (**e**), respectively. (**i**,**j**) The fate transformation phenotypes were quantified as a percentage of worms after the L2.2 and the L3 asymmetric divisions, by dividing the number of worm exhibiting each phenotype by the total number of worms analysed.

Having examined its involvement in physical division asymmetry, we next tested whether *nmy-2* is involved in downstream cell fate specification instead. A dual-colour fate reporter strain was constructed to examine whether *nmy-2* knockdown causes cell fate mis-specification. This strain marks the seam fate (retained by the posterior daughters) with a red fluorophore (*scmp::Tomato*) (Fig. 3a, lollipop arrow) and the hypodermal fate (adopted by anterior daughters) with a green one (*dpy-7p::YFP*) (Fig. 3a, diamond arrow). As a control, the fate reporter strain was used to illustrate the normal hypodermal differentiation event in the anterior daughters following an asymmetric division (Supplementary Fig. S6). The anterior daughters first expressed the red seam marker whilst rounding up, and then switched to expressing the green hypodermal marker when still in the seam line, before moving out of the line and fusing with the hypodermis. This sequence of events illustrates that a fate change can indeed be visualised by a colour change using this reporter strain.

Three distinct classes of cell fate mis-specification were identified in *nmy-2* knockdown animals following asymmetric seam cell divisions. Class I was a direct seam-to-hypodermal fate change. Cells that should exclusively express the red seam marker were observed inappropriately expressing the green hypodermal marker whilst retaining a low level of red signal (Fig. 3b, arrows; cell in inset box (arrowhead) shown in c (red channel, arrowhead) and d (green channel, arrowhead)), suggestion a fate transition. Class II and III both involved bi-nucleated cells, with both nuclei expressing either the green hypodermal marker in Class II (Fig. 3e, arrow) or the red seam marker in Class III (Fig. 3h, arrow). These bi-nucleated cells arose from cytokinesis defects and were common in *nmy-2* knockdown given the essential role of *nmy-2* in cytokinesis. Bi-nucleated cells could also be captured in the process of acquiring a different fate, as both the red and the green markers were simultaneously expressed (Fig. 3e, cell in inset box (arrowhead) shown in f (red channel, arrowhead) and g (green channel, arrowhead)).

**Figure 4:**
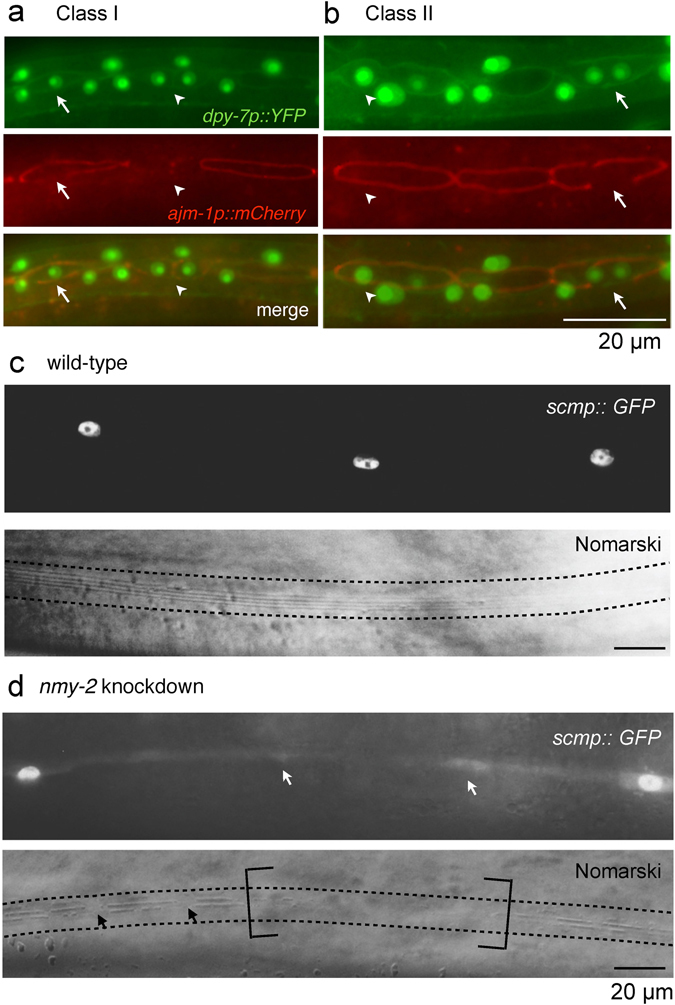
Class I and II fate transformations resemble normal hypodermal differentiation. (**a**,**b**) Worms with the dual-colour fate reporter *ouIs10* and the integrated apical junction marker *ouIs21* (strain AW1106) were subjected to *nmy-2* knockdown and representative snapshots were taken after the L3 division. (**a**) Class I phenotype. Arrow points to the broken apical junction in a seam daughter that should normally be protected from receiving differentiation signals. Arrowhead points to a Class II event next to a Class I event in the same worm. (**b**) Class II phenotype. Arrow points to the broken apical junction in a seam daughter that should normally be protected from receiving differentiation signals. Arrowhead points to a cell at the transitional stage where the hypodermal marker is expressed more strongly in the anterior nucleus than the posterior one. (**c**) Wild-type seam syncytium produced smooth, continuous alae (between dotted lines) (strain AW335). (**d**) The *nmy-2* knockdown animal had missing seam nuclei (white arrows) that correlated with absent (brackets) or broken (black arrows) alae ridges (strain AW785). Young adults were imaged in (**c**) and (**d**).

Quantification of the phenotypes showed that *nmy-2* inactivation led to Class I, II or III phenotypes in 43%, 59% or 19% of the worms after the L2.2 division, respectively (Fig. 3i), and in 43%, 61% or 55% of the worms after the L3 division, respectively (Fig. 3j). The penetrance was similar between the L2.2 and L3 asymmetric division datasets except for Class III, which was more common after the L3 division. Because it was possible to have multiple phenotype classes (Fig. 4a: arrow, Class I; arrowhead, Class II) and/or multiple events of the same class in a single worm, the phenotypes were also quantified as the percentage of total V lineage cells (Supplementary Fig. S7), which revealed a similar pattern of penetrance.

In summary, *nmy-2* knockdown produced three classes of cell fate mis-specification phenotypes. Since Class I and II phenotypes (leading to seam nuclei loss) were more common than Class III (leading to seam nuclei gain), the quantification data were consistent with the overall reduction in the number of seam nuclei at adulthood that we observed.

### Class I and II fate transformations feature normal differentiation in the wrong cells

As the predominant Class I and II phenotypes involved a seam-to-hypodermal transformation, we next tested the extent to which these switches in fate markers resemble the normal anterior differentiation event occurring in the wrong daughter cell. We have previously reported that apical junction disintegration precedes hypodermal fate acquisition in the anterior daughter^41^, a process that depends on the fusogen EFF-1^42, 43^. EFF-1 punctures the cell membrane^44^ to allow anterior daughters to receive differentiation signals from the surrounding hyp7 syncytium. An integrated apical junction marker (*ajm-1p::mCherry*) was crossed into the dual-colour fate reporter strain, and compromised apical junctions were indeed found to be associated with both Class I and II phenotypes (Fig. 4a,b, arrows). This suggests that these seam-to-hypodermal fate transformations allow differentiation events to occur in cells that would normally retain the seam fate of further proliferation. Further evidence for this inappropriate differentiation of posterior daughters were the gaps in the integrity of the alae that could be observed in adult animals following *nmy-2* knockdown (Fig. 4d). Secretion of alae is the normal terminal fate of seam cells (Fig. 4c) but was never observed in *dpy-7* positive hypodermal cells, suggesting cell fate switching in these cells.

Bi-nucleated cells displaying the class II phenotype in which cytokinesis had failed could be captured at a transitional stage, in which the anterior nucleus expressed the hypodermal marker more strongly than the posterior one (Fig. 4b, arrowhead). Reversed hypodermal marker asymmetry (i.e. the posterior nucleus showing stronger hypodermal expression than the anterior nucleus) was never observed in *nmy-2* knockdown, indicating that the division polarity was unaffected as it was the anterior compartment that underwent normal differentiation first. Based on these observations, it seems likely that the anterior hypodermal signal permeated through the rest of the cell due to improper cytoplasm segregation, thereby inducing the posterior nucleus to turn hypodermal with a slight delay with respect to the anterior one.

### *nmy-2* regulates cell fate determination independently of cytokinesis

**Figure 5:**
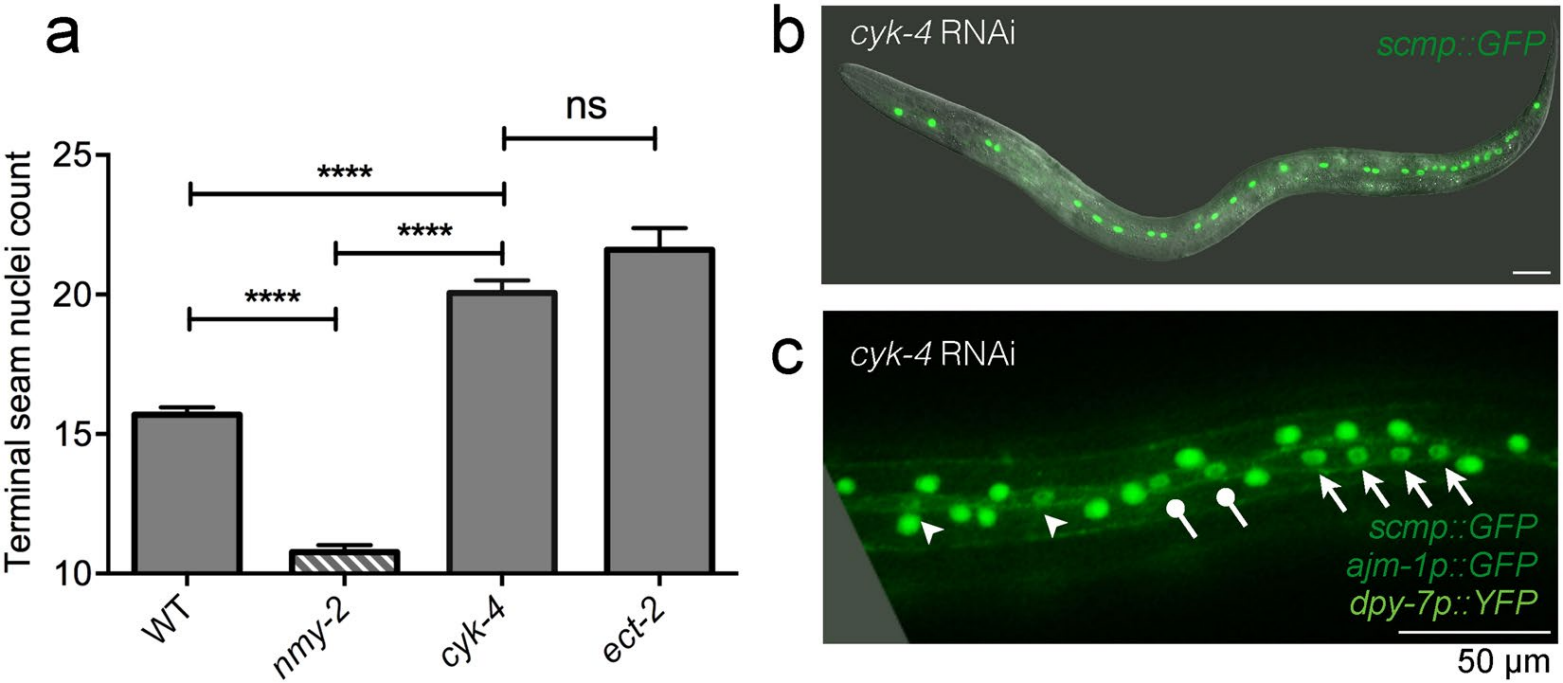
Disrupting cytokinesis alone increases terminal seam nuclei count. (**a**) Post-embryonic *cyk-4* and *ect-2* RNAi produced extra terminal seam nuclei. Synchronised L1 larvae were fed the respective RNAi bacteria at 25 °C and terminal seam nuclei were counted in young adult hermaphrodites using the *wIs51* seam cell marker. n > 50 for each sample. Error bars represent the SEM. Unpaired, two-tailed *t*-test was used to determine significance (****p < 0.0001; ns: p = 0.09). Two independent replicates of the experiment were performed. (**b**,**c**) Representative images of *cyk-4* RNAi adults showing (**b**) seam hyperplasia (strain JR667) and (**c**) unevenly spaced terminal seam nuclei (strain AW1147). Arrows and lollipop arrows point to nuclei that cluster together, and arrowheads point to nuclei that are more sparsely distributed. The phenotypes were virtually identical between *cyk-4* and *ect-2* RNAi, so the *ect-2* data are omitted for simplicity.

Cytokinesis failure was common in *nmy-2* knockdown, raising the possibility that all classes of *nmy-2*-associated cell fate defects are a secondary consequence of failed cytokinesis. To test this, we assessed whether other known regulators of cytokinesis resulted in a similar phenotype. CYK-4 (a component of the central spindle) and ECT-2 (a guanine nucleotide exchange factor required for the activation of RhoA small GTPase) are two upstream activators of the RhoA-dependent cytokinesis pathway (while NMY-2 is a downstream effector) and are both absolutely required for cytokinesis. ECT-2 localises to the central spindle by binding to CYK-4; at anaphase onset ECT-2 translocates to the equatorial cell cortex to activate RhoA, leading to actomyosin assembly to initiate furrow ingression^45–47^. Strikingly, post-embryonic RNAi of either *cyk-4* or *ect-2* produced the complete opposite effect to *nmy-2* knockdown with respect to seam cell number. Whereas *nmy-2* perturbation caused an overall reduction of terminal seam nuclei, *cyk-4* or *ect-2* RNAi led to an overall increase (Fig. 5a,b). On average, *cyk-4* and *ect-2* RNAi resulted in 20.0 and 21.6 terminal seam nuclei per side, respectively. The animals exhibited uneven seam nuclei spacing, with some nuclei clustering together (Fig. 5c, arrows and lollipop arrows each pointing to one cluster) compared to the others (Fig. 5c, arrowheads). This is in contrast to the evenly spaced nuclei in the wild-type seam syncytium (Fig. 2b). The nuclei clusters may have arisen from polyploid cells as a consequence of failed cytokinesis before terminal seam fusion.

This experiment allows us to uncouple defects in cell fate allocation from defects caused by cytokinesis alone. *nmy-2* must have additional functions in the seam, such that the overall effect of *nmy-2* perturbation is a net decrease of seam cells.

### Interaction of *nmy-2* with other known seam regulatory components

**Figure 6:**
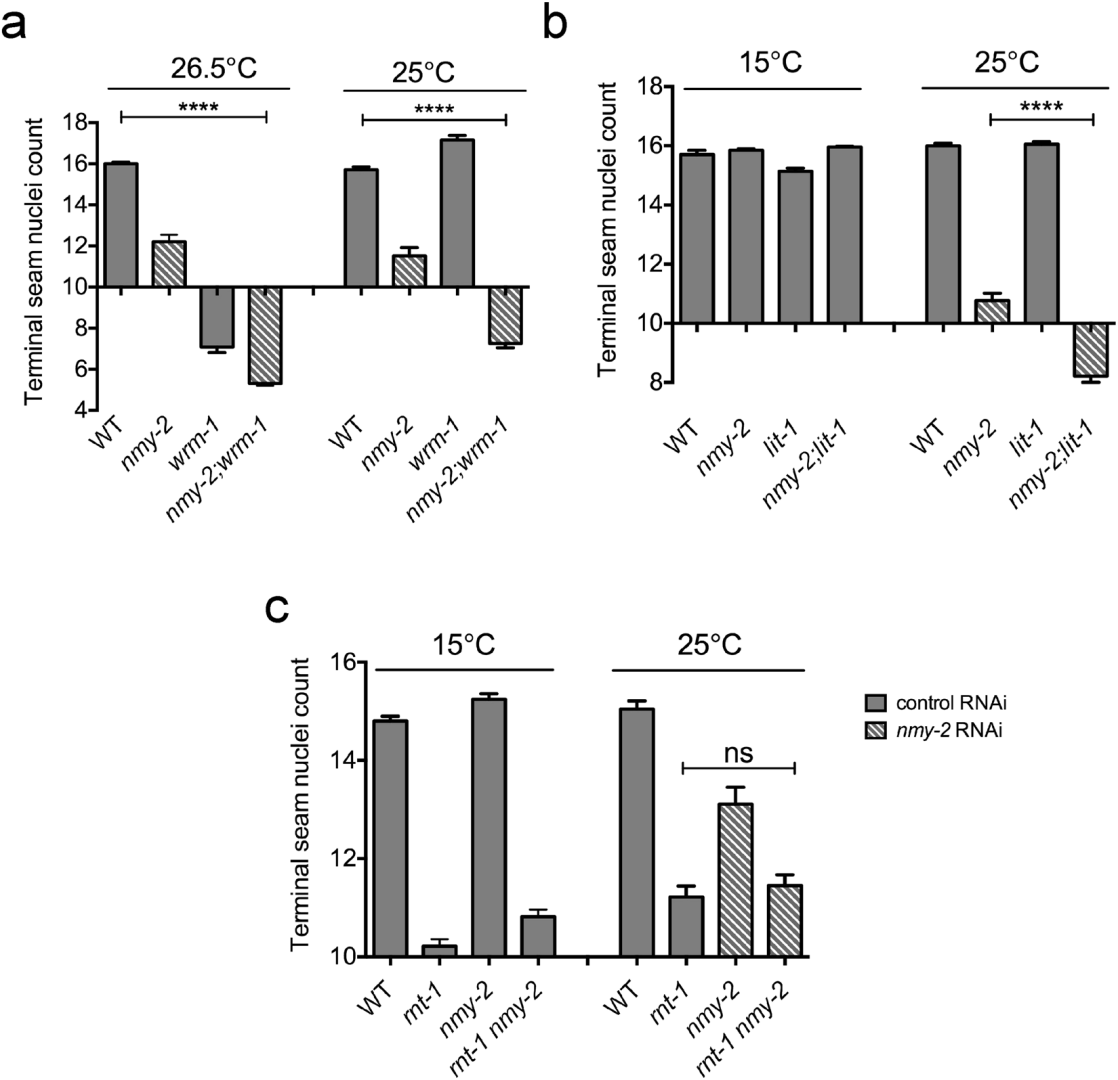
Epistasis analysis between *nmy-2* and known seam regulators. (**a**) *nmy-2* and *wrm-1*. Synchronised L1 larvae for the wild-type, *nmy-2* and *wrm-1* single mutants, and the *nmy-2*; *wrm-1* double mutant (strains AW335, AW785, EW95 and AW861, respectively) were fed the indicated RNAi bacteria at 26.5 °C or 25 °C. (**b**) *nmy-2* and *lit-1*. Synchronised L1 larvae for the wild-type, *nmy-2* and *lit-1* single mutants, and the *nmy-2;lit-1* double mutant (strains AW335, AW785, AW943 and AW983, respectively) were fed the indicated RNAi bacteria at 15 °C or 25 °C. (**c**) *nmy-2* and *rnt-1*. Synchronised L1 larvae for the wild-type, *rnt-1* and *nmy-2* single mutants, and the *rnt-1 nmy-2* double mutant, all in the *dpy-5* background (strains AW989, AW990, AW991 and AW992, respectively), were fed the indicated RNAi bacteria at 15 °C or 25 °C. Terminal seam nuclei were counted in young adult hermaphrodites using the *wIs51* seam cell marker. n > 50 for each sample. Error bars represent the SEM. Unpaired, two-tailed *t*-test was used to determine significance (****p < 0.0001; ns: p = 0.46). Three independent replicates of the experiment were performed.

In order to investigate the molecular mechanism by which loss of *nmy-2* causes seam to hypodermal cell fate transformations, we investigated whether it interacts with the Wnt/β-catenin asymmetry pathway. A double mutant was constructed, containing both the *nmy-2*(*ne3409ts*) allele and the *wrm-1*(*ne1982ts*) allele, the latter of which is known to have little post-embryonic effect at 25 °C but significantly reduce seam cell number at 26.5 °C^10, 18^. Indeed, at 26.5 °C, perturbing *wrm-1* either in isolation or in combination with *nmy-2* both reduced seam nuclei count to a similar extent, presumably due to the strong masking effect of the *wrm-1* mutation (Fig. 6a). The 25 °C dataset was more useful in demonstrating that seam nuclei loss was enhanced in the double mutant (Fig. 6a). Double mutant analysis was repeated using *nmy-2* and *lit-1*(*or131ts*), a second Wnt pathway component that works together with WRM-1, and similar results were obtained (Fig. 6b).

The enhanced phenotype of the *nmy-2*;*wrm-1* double mutant was observed not only during seam development but also during embryogenesis, as the permissive temperature for embryonic viability was lowered from 20 °C in either single mutant to 17 °C in the double mutant (Supplementary Fig. S8). This suggests that *nmy-2* likely functions in parallel to *wrm-1* during embryonic development as well as post-embryonically during seam development.

As loss of the RNT-1 transcription factor is known to result in a similar seam cell loss phenotype to *nmy-*2 (and *rnt-1* is also thought to act in parallel to Wnt signalling^10^), we next examined whether *nmy-2* enhanced the *rnt-1* phenotype. A double mutant between *nmy-2* and *rnt-1*(*e1241*) was constructed. *rnt-1*(*e1241*) behaves as a null allele that decreased the terminal seam nuclei count from 14.8–15.0 per side (in a *dpy-5* mutant background, which was included for ease of crossing) to 10.2–11.2 per side due to proliferation defects, at both 15 °C and 25 °C (Fig. 6c). *nmy-2* perturbation did not enhance this phenotype at either temperature, suggesting that *rnt-1* and *nmy-2* potentially function in the same pathway.

## Discussion

In this study, we investigated the role of *nmy-2* in *C. elegans* seam cell asymmetric divisions. We found that knocking down *nmy-2* post-embryonically using a combination of RNAi and ts mutations caused progressive seam cell loss after asymmetric divisions. Seam cells are lost through inappropriate seam-to-hypodermal cell fate transformations when *nmy-2* function is disrupted, suggesting that *nmy-2* is required for correct cell fate specification following division. We could uncouple the role of *nmy-2* in cytokinesis from its role in cell fate determination, as disrupting other cytokinesis pathway components was not observed to lead to seam cell loss.

Close examination of cell fate distribution in bi-nucleated cells resulting from failed cytokinesis in *nmy-2* knockdown animals highlights the effectiveness of the hypodermal differentiation signal in promoting differentiation of every nucleus it reaches. We never observed a bi-nucleated cell where one nucleus expressed the differentiation marker while the other stably expressed the seam marker. Thus, anterior daughter differentiation must normally be tightly coordinated with the timing of cytokinesis to ensure that the powerful differentiation signal, presumably necessary to ensure phenotypic uniformity in the hyp7 syncytium formed by the fusion of anterior seam-derived daughters, is kept away from the posterior self-renewing seam daughter.

We found no obvious role for *nmy-2* in regulating physical seam cell division asymmetry, as the positioning of the chromosome and the cleavage site appeared to be unaltered by *nmy-2* knockdown. Rather, *nmy-2* appears to influence cell fate patterning independently of this. Nevertheless, the wild-type size data (Supplementary Fig. S4), which have never been previously reported, reveal some intriguing aspects of seam biology. Firstly, we found that the nucleus is often displaced away from the centre of the cell. This is observed in various polarised cells, although the biological role of this off-centre placement is mostly unknown. An example of a biologically relevant nuclear displacement is the early embryonic EMS division, where the posterior but not anterior daughter nucleus is anchored to the posterior cell cortex by centrosomes. This nuclear anchoring is controlled by Wnt and Src signalling in the EMS cell and affects the asymmetric nuclear localization of POP-1^48^. The regulation and relevance of seam cell nuclear displacement and the subsequent spindle displacement is not clear, however the data presented here suggest that they do not dictate the fate outcome of seam cell divisions.

Moreover, we observed off-centre placement of the wild-type L2.2 division cleavage furrow. This could represent a physical mechanism to switch from the symmetric to the asymmetric mode of division. Unlike the other more “stereotypical” seam cell asymmetric divisions, the L2.2 asymmetric division is unique in that it immediately follows the L2.1 symmetric division. Perhaps due to time and space constraint, the L2.2 division needs to displace the cleavage furrow to antagonise or override L2.1 “symmetrical division” instructions in order to generate physical division asymmetry for the L2.2 division. Two other cases of displaced cleavage furrow have been reported as important for the asymmetric divisions of the *Drosophila* neural stem cells^34, 35^ and the *C. elegans* Q.a neuroblast^33^. While both of these systems rely on polarised myosin II for displacement, *nmy-2* appears dispensable for displacing the furrow during the L2.2 division of *C. elegans* seam cells.

The role of *nmy-2* in influencing cell fate determination during seam development may involve interaction with known seam cell regulatory factors such as RNT-1, which appears epistatic to *nmy-*2. It is conceivable that *nmy-2* may function upstream of RNT-1, perhaps by asymmetrically trafficking RNT-1 pathway components such as CEH-20 or RNT-1 itself, both of which show asymmetric localisation in the daughters^18, 19, 21^. A precedent for the role of *nmy-2* in trafficking was established in the *C. elegans* zygote, where anteriorly-restricted actomyosin contractility generates polarised cytoplasmic flow that transports anterior PAR proteins to the appropriate cytoplasmic location^25, 27–29^. The fibre-like structures formed by NMY-2 punctae (Fig. 1f) may implicate contraction in the A-P orientation to transport cell fate determination factors. However, we did not observe preferential localisation of NMY-2 to either the anterior or posterior daughter at or immediately after division, in contrast to what has been observed in the Q neuroblasts. It is also curious that neither *nmy-2* allele affecting the S2 region had any effect on post-embryonic seam cell divisions, suggesting that the motor function of NMY-2 may not actually be required for its role in post-embryonic seam cell divisions.

The data presented here suggest that *nmy-2* function is required for correct cell fate specification, but not physical division asymmetry, of seam cell divisions. In a broader context, our results support a general role for *nmy-2* in regulating *C. elegans* asymmetric divisions during both embryonic (zygote, EMS) and post-embryonic (Q.a neuroblast, seam cells) development, in several different lineages. The mechanism by which *nmy-2* exerts regulation, however, varies for each system. In the zygote and Q.a neuroblast, *nmy-2* affects polarity establishment and daughter cell sizes, respectively, both of which concern the physical division asymmetry. By contrast, in both EMS and the seam cells, *nmy-2* may regulate differential fate specification independently of physical asymmetry. In the case of EMS, *nmy-2* functions in Src-dependent phosphotyrosine signalling to specify the endoderm fate in the E daughter of the division in parallel to Wnt signalling (consistent with the synthetic embryonic lethality we observed between *nmy-2* and *wrm-1* (Supplementary Fig. S8)). In our study, seam cells present an intriguing comparison whereby *nmy-2* similarly acts in parallel to Wnt to specify appropriate daughter fates in the seam lineage, in addition to preventing inappropriate cell fate transformations by effectively executing cytokinesis. In summary, our findings contribute to the notion that *nmy-2* regulates asymmetric divisions in a number of cell lineages in a wide range of organisms using diverse mechanisms.

## Materials and Methods

### *C. elegans* strains

*C. elegans* strains were derived from the wild-type Bristol N2 reference strain or the CB4856 Hawaiian strain. Worms were maintained by standard methods^49^. Strains were grown on 55 mm Nematode Media Growth (NGM) plates and fed *E. coli* strain OP50, at room temperature or at 15 °C if they were ts. The strains used in this study are listed in Supplementary Table 1.

### Live fluorescent microscopy

A low power Leica MZIII fluorescent dissecting microscope (1–10x objective) was used to visualise bright fluorescence without the need for mounting, as was necessary for crossing in fluorescent cellular markers, for example.

To visualise phenotypes in greater detail, a Zeiss Axiophot DIC/fluorescent microscope or a Leica SP5 laser scanning confocal microscope (10–63x objective) was used. Live worms were mounted onto 2% agarose pads and anesthetised in 0.5% phenoxypropanol (Aldrich) before being visualised. The Zeiss microscope is equipped with an AxioCam camera and AxioVision software for image acquisition.

### Molecular cloning

Full-length *nmy-2* promoter was cloned into the pPD107.94 enhancer assay plasmid (Addgene #1531) to produce pAW850, so that transgenic worms could be generated to assess when and where the promoter drives transcription. *nmy-2* full-length promoter was defined as the 1723-bp region between the end of the last gene preceding *nmy-2* and the *nmy-2* start codon. The promoter was amplified from N2 genomic DNA using the nmy-2p-F (5′-TATCTGAAATTTGAAAAAAAAGTGAGTTATTTTTTTCG-3′) and nmy-2p-R (5′-TATTACCGCTGGAGCTGTTGTTGTAATCA-3′) primer pair. AccuPrime Taq DNA polymerase (Invitrogen) was used for the PCR reaction and the product was cloned into TOPO XL vector (Invitrogen) in the forward orientation to generate pAW849. pAW849 and pPD107.94 vector were digested with XbaI and HindIII. Appropriate fragments were isolated and ligated together to produce pAW850, which contains the *nmy-2* full-length promoter inserted between the HindIII and XbaI sites of pPD107.94. Unfortunately a small region of the promoter could not be sequenced, probably due to the formation of a hairpin structure.

### Construction of transgenic animals

Transgenic animals were made by microinjection as described^50^ using *unc-119* as a transformation marker. Plasmids were injected at 2–20 ng/μL. Stable transgenic lines were isolated and maintained at 25 °C for several generations before being routinely maintained at room temperature.

When necessary, extra-chromosomal arrays were integrated into the *C. elegans* genome by gamma irradiation from a cesium source at the Sir William Dunn School of Pathology, University of Oxford. *unc-119*-rescued lines with medium transmission rate (30–60%) were selected for integration. Seventy L4 worms were exposed to 40 Gy of irradiation and 350 healthy F1’s were isolated 3–4 days later. Twenty-five F1’s that gave 75–100% transmission rate were further isolated and clonally expanded for 2–4 generations to achieve stable transgene integration that resulted in 100% non-Unc lines. Independent lines were outcrossed 4–6 times to remove background mutations before being used for further analysis.

### RNAi by feeding

RNAi experiments were performed by feeding worms HT115 bacteria expressing double-stranded RNA corresponding to the target gene. The Ahringer RNAi library^51^ clones (Source BioSciences) were used for all the experiments. Glycerol stocks of the clone were streaked onto a 2xYT plate supplemented with 50 μg/mL ampicillin, from which a 2 mL liquid culture was grown in 2xYT Amp broth at 37 °C for 6–18 h. RNAi plates (modified from regular NGM to include 1 mM IPTG and 25 ng/mL carbenecillin) were seeded with 120 μL of the overnight culture and inoculated at room temperature overnight before worms were placed. RNAi experiments took place at 25 °C unless otherwise indicated. HT115 bacteria transformed with empty L4440 vector was used as the negative control (“control RNAi”) and *pop-1* L4 RNAi with the embryonic lethal phenotype was used as the positive control.

In order to bypass any embryonic effect, nearly all RNAi experiments in this study were conducted directly on L1 larvae to assess the worms at a later developmental stage (post-embryonic RNAi). This was done by spot bleaching ~30 gravid hermaphrodites onto an RNAi plate to examine a relatively small number of progeny that hatched from the bleach-resistant eggs. If a larger number of affected worms were desired, then multiple plates of gravid hermaphrodites were bleached and their progeny synchronised to the L1 diapause before being transferred to RNAi plates. In Supplementary Fig. S2 only, RNAi was performed on L4 larvae for assessing their progeny (L4 RNAi). For L4 RNAi, three to five L4 larvae were placed onto seeded RNAi plates and incubated at 25 °C until their progeny developed to the desired stage for examination.

### Bleach synchronisation

Worms were synchronised by bursting gravid mothers with bleach to release the more bleach-resistant eggs. Briefly, gravid worms were washed off of two to five 90 mm NGM plates with M9 buffer and washed several times to remove bacteria. 4 mL of 2x bleach solution (1 M NaOH, 4.8% sodium hypochlorite) was added to 4 mL of worm pellet in M9 in a 15 mL Falcon tube. The tube was vortexed vigorously for 4 min to burst the gravid worms and topped up with M9 immediately after vortexing. Eggs were pelleted by centrifugation at 560 *g* for 1.5 min. The egg pellet was quickly washed several times to remove any residual bleach and allowed to hatch overnight on a rotator in M9, at room temperature or at 15 °C if the strains were ts. To re-feed synchronised, starved L1s, worms were pelleted by centrifugation at 560* g* for 1.5 min and examined in a watch glass before being transferred onto plates containing OP50 or RNAi bacteria and placed at appropriate temperatures.

### Genotyping

Single worms were lysed to release genomic DNA before being used for genotyping PCR. Briefly, individual worms were picked into 2.5 μL of worm lysis buffer (50 μM KCl, 2.5 μM MgCl2, 10 μM Tris-HCl pH 8.3, 0.45% NP40, 0.45% Tween-20, 0.01% gelatine, 0.1 mg/ml proteinase K) in the lid of a 0.5 mL Eppendorf tube and then briefly spun down to the bottom. The tube was frozen at −80 °C for 15 min, followed by incubation in a PCR machine at 60 °C for 1 h and then at 95 °C for 15 min. This lysis mix was stored at −20 °C or used immediately as the DNA template. It was possible to perform two sets of genotyping PCR from the same lysis mix by splitting the 2.5 μL template in half.

Genotyping PCRs were carried out to track mutant alleles that do not produce a unique visible phenotype. For *nmy-2*(*ne3409*), *nmy-2*(*ne1490*), *wrm-1*(*ne1982*), *lit-1*(*or131*) and *rnt-1*(*e1241*) missense mutations, tetraARMS PCR primers were designed as described^52^ to follow the mutant alleles. Additionally, GFP genotyping was used to track GFP-tagged cellular markers during a cross if the markers were invisible on the low power Leica dissecting microscope. GFP genotyping was achieved using the GFP-F (5′-GGAGAAGAACTTTTCACTGGAGTTGTCC-3′) and GFP-R (5′-CCATGCCATGTGTAATCCCAGCAGCTGT-3′) primer pair, which gives a 844-bp band following a standard PCR reaction.

### Statistical analysis

Statistical analysis was performed using GraphPad Prism (Version 6.0 g). Unpaired, two-tailed *t*-tests were used to determine significance between two groups of data in the same set of experiment unless otherwise indicated.

## Electronic supplementary material

Supplementary Information
