# Supplementary material for: Non-muscle myosin II is required for correct fate specification in the Caenorhabditis elegans seam cell divisions
Source: Sci Rep. 2017 Jun 14;7:3524. doi: 10.1038/s41598-017-01675-7 (PMC5471188; doi:10.1038/s41598-017-01675-7)
Supplement: Supplementary file 1 — Supplementary Information [file 41598_2017_1675_MOESM1_ESM.pdf]

Non-muscle myosin II is required for correct fate specification in the *Caenorhabditis elegans* seam cell divisions

Siyu Serena Ding[1,2,3] & Alison Woollard[1]*

[1] Department of Biochemistry, University of Oxford, South Parks Road, Oxford OX1 3QU, United Kingdom

[2] Present address: Institution of Clinical Sciences (ICS), Faculty of Medicine, Imperial College London, Du Cane Road, London W12 0NN, United Kingdom

[3] Present address: MRC London Institute of Medical Sciences (LMS), Hammersmith Hospital Campus, Du Cane Road, London W12 0NN, United Kingdom

* Correspondence should be sent to alison.woollard@bioch.ox.ac.uk

Supplementary Tables

Supplementary Table 1. *C. elegans* strains used in this study

Strain Name	Genotype
AW1118	*unc-119(ed3)III;ouEx849[nmy-2p::GFP(pAW850)+unc-119(+)]*
AW1092	*nmy-2(cp13[nmy-2::GFP+LoxP])I;ouIs21[ajm-1p::mCherry]*
AW335	*wIs51[scmp::GFP] him-5(e1490)V*
AW785	*nmy-2(ne3409)I;wIs51[scmp::GFP] him-5(e1490)V*
AW786	*nmy-2(ne1490)I;wIs51[scmp::GFP] him-5(e1490)V*
SV1009	*heIs63[wrt-2p::GFP::PH,wrt-2p::GFP::H2B,lin-48p::mCherry]*
AW788	*nmy-2(ne3409)I;him-5(e1490)V;heIs63[wrt-2p::GFP::PH,wrt-2p::GFP::H2B,lin- 48p::mCherry]*
AW1015	*ouIs10[scmp::NLS::tdTomato(pAW584)+wrt-2p::GFP::PH(pAW561)+dpy-7p::2xNLS::YFP(pAW516)]*
AW1018	*nmy-2(ne3409)I;ouIs10[scmp::NLS::tdTomato+wrt-2p::GFP::PH+dpy-7p::2xNLS::YFP]*
JR667	*unc-119(e2498::Tc1)III;wIs51[scmp::GFP]V*
AW1147	*wIs51[scmp::GFP];syIs78[ajm-1p::GFP];arIs99[dpy-7p::2xNLS::YFP]*
EW95	*wrm-1(ne1982)III;wIs51[scmp::GFP]V*
AW861	*nmy-2(ne3409)I;wrm-1(ne1982)III;wIs51[scmp::GFP] him-5(e1490)V*
AW943	*lit-1(or131)III;wIs51[scmp::gfp] him-5(e1490)V*
AW983	*nmy-2(ne3409)I;lit-1(or131)III;wIs51[scmp::gfp] him-5(e1490)V*
AW989	*dpy-5(e61) rnt-1(e1241) nmy-2(ne3409)I;wIs51[scmp::GFP] him-*
AW990	*dpy-5(e61) rnt-1(e1241)I;wIs51[scmp::GFP] him-5(e1490)V*
AW991	*dpy-5(e61), nmy-2(ne3409)I;wIs51[scmp::GFP] him-5(e1490)V*
AW992	*dpy-5(e61)I;wIs51[scmp::GFP] him-5(e1490)V*
AW1120	*unc-119(ed3)III;ouEx851[nmy-2p::GFP(pAW850)+unc-119(+)]*
AW1095	*heIs63[wrt-2p::GFP::PH,wrt-2p::GFP::H2B,lin-48p::mCherry];ouIs21[ajm-1p::mCherry]*
AW1116	*nmy-2(ne3409)I;heIs63[wrt-2p::GFP::PH,wrt-2p::GFP::H2B,lin-48p::mCherry];ouIs21[ajm-1p::mCherry]*

Supplementary Figures

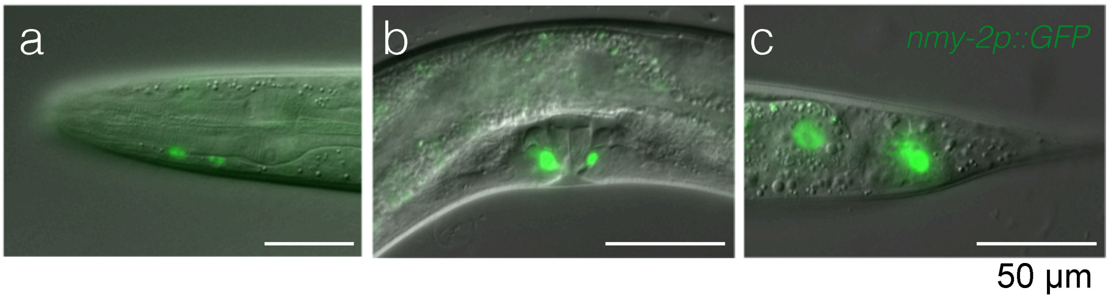

Supplementary Figure S1. *nmy-2* is expressed in various tissues other than the seam in adults. *nmy-2* transcriptional reporter (strain AW1120) showed expression in the **a**) head neurons, **b**) vulva, and **c**) posterior intestine and the rectal gland.

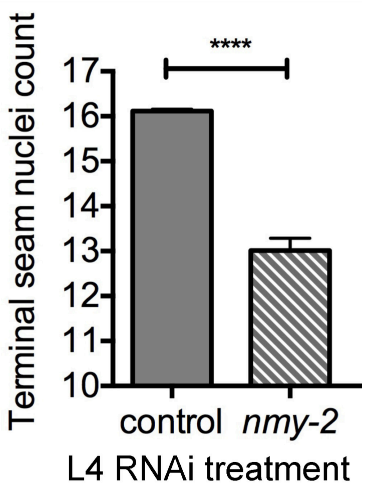

Supplementary Figure S2. RNAi treatment at the L4 stage reduces the number of seam cells. Wild-type L4 larvae with the integrated seam cell marker *wIs51[scmp::GFP]* (strain JR667) were fed control or *nmy-2* RNAi at 25°C and seam nuclei were counted in their progeny at the young adult stage. n>50 for each sample. Error bars represent the standard error of the mean (SEM). Unpaired, two-tailed *t*-test was used to determine significance (****: p<0.0001). At least three independent replicates of the experiment were performed.

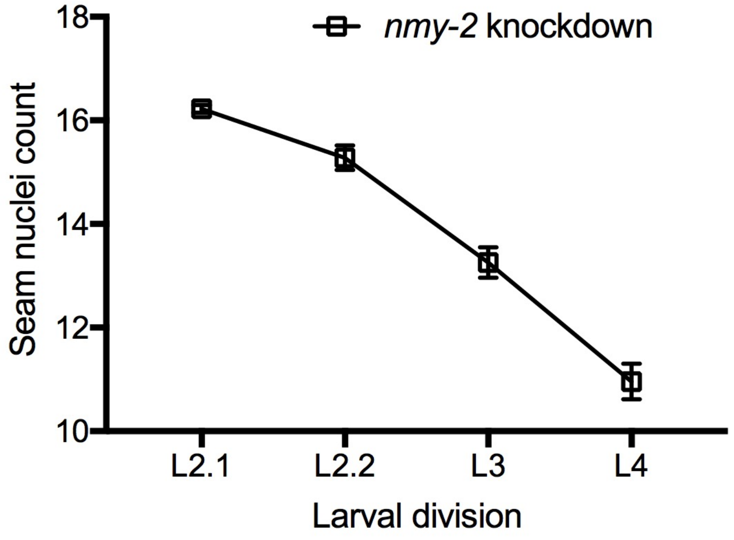

Supplementary Figure S3. Seam nuclei numbers decrease after each asymmetric division following *nmy-2* knockdown. *nmy-2* inactivation data from Fig. 2f are represented here as seam nuclei count, using the same imaging dataset to generate both figures. The number of seam nuclei were counted using the integrated DNA marker *wrt-2p::gfp::H2B* (strain AW788). L2.1 = L2 first (symmetric) division. L2.2 = L2 second (asymmetric) division. n>50 per strain per stage. Error bars represent the standard error of the mean (SEM). Two independent replicates of the experiment were performed.

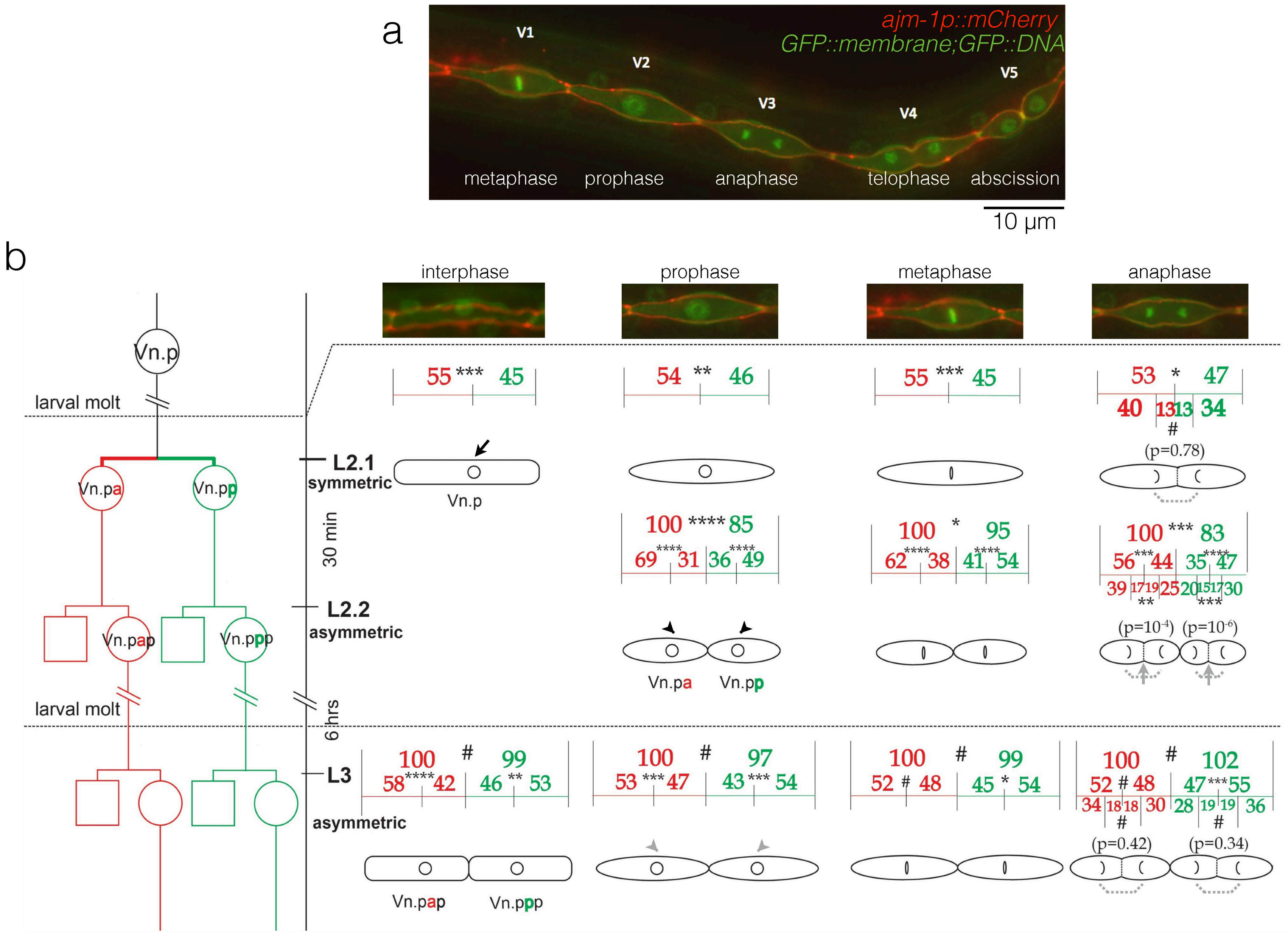
a
ajm-1p::mCherry
GFP::membrane;GFP::DNA
V1
V2
V3
V4
V5
metaphase
prophase
anaphase
telophase
abscission
10 µm
b
interphase
prophase
metaphase
anaphase
Vn.p
larval molt
Vn.pa
Vn.pp
Vn.pap
Vn.ppp
L2.1
symmetric
30 min
L2.2
asymmetric
6 hrs
L3
asymmetric
(p=0.78)
(p=10-4)
(p=10-6)
(p=0.42)
(p=0.34)

Supplementary Figure S4. Wild-type chromosome and furrow positioning during seam cell divisions. **a**) Representative stages measured to generate the dataset. The worm is undergoing the L1 asymmetric division. **b**) Strain AW1095 was bleach synchronised to L1 and transferred to OP50 plates at 25°C. Detailed methods for quantitative measurements can be found in Supplementary Methods. Squares and circles represent hypodermal and seam cells, respectively; red and green texts indicate the anterior and posterior branch lineages following the L2.1 symmetric division, respectively. Figures are drawn to scale in the A-P orientation within each division. All measurements within a cell were normalised to the full A-P length of that cell, which was assigned a value of 100 (e.g. The first number in the first row, 55, indicates that in the L2.1 interphase cell, the distance between the anterior end of the cell and the centre of the nucleus corresponds to 55% of the total A-P cell length. Likewise, the last two numbers in the first row, 13 and 34, indicate that in the L2.1 anaphase cell, the distances between the cleavage furrow and the posterior set of chromosome, and between the posterior chromosome and the posterior end of the cell, are 13% and 34% of the total A-P cell length, respectively). Vertical timelines indicate the approximate time between each division at 25°C. n>45 for each stage of each division. Two-tailed *t*-tests were performed to determine significance (#: $p>0.05$; *: $p<0.05$; **: $p<0.01$; ***: $p<10^{-5}$; ****: $p<10^{-10}$). There are four trends. First, nuclear placement prior to division does not dictate the daughter fate outcome. The symmetric division (L2.1) initiates with a displaced nucleus (black arrow) and generate two proliferative daughters Vn.pa and Vn.pp; likewise the asymmetric division (L2.2) starts out with the nuclei (black arrowheads) closer

to either one or other end of the cell and invariably produce a differentiating anterior daughter and a proliferative posterior daughter. Secondly, the nuclei in the L2.1-derived anterior (red) versus posterior (green) branch daughters are paired across divisions. Vn.pa and Vn.pp nuclei (black arrowheads) are displaced towards the centre of the pair as if they were the “crossed” irises of two eyes. This pairing persists in L3 between Vn.pap and Vn.ppp (gray arrowheads) despite hours of quiescence and the L2/L3 larval moult, suggesting that the nuclear pairing is likely a result of branch identity. Thirdly, there is a lack of overt spindle displacement (gray dashed brackets) as the main mechanism for generating different daughter sizes following seam cell divisions. The rapid re-sizing and re-shaping of the daughters appear to primarily take place post-division. Finally, while the cleavage furrow perfectly bisected segregating chromosomes in the L2.1 and L3 divisions, during L2.2 anaphase it was displaced more towards the anterior set of chromosomes in both the Vn.pa and the Vn.pp cells (gray arrows). Because L2.2 uniquely occurs immediately after another division, this L2.2-specific furrow displacement could represent a mechanism for generating physical division asymmetry under temporal and spatial constraints following the symmetric division.

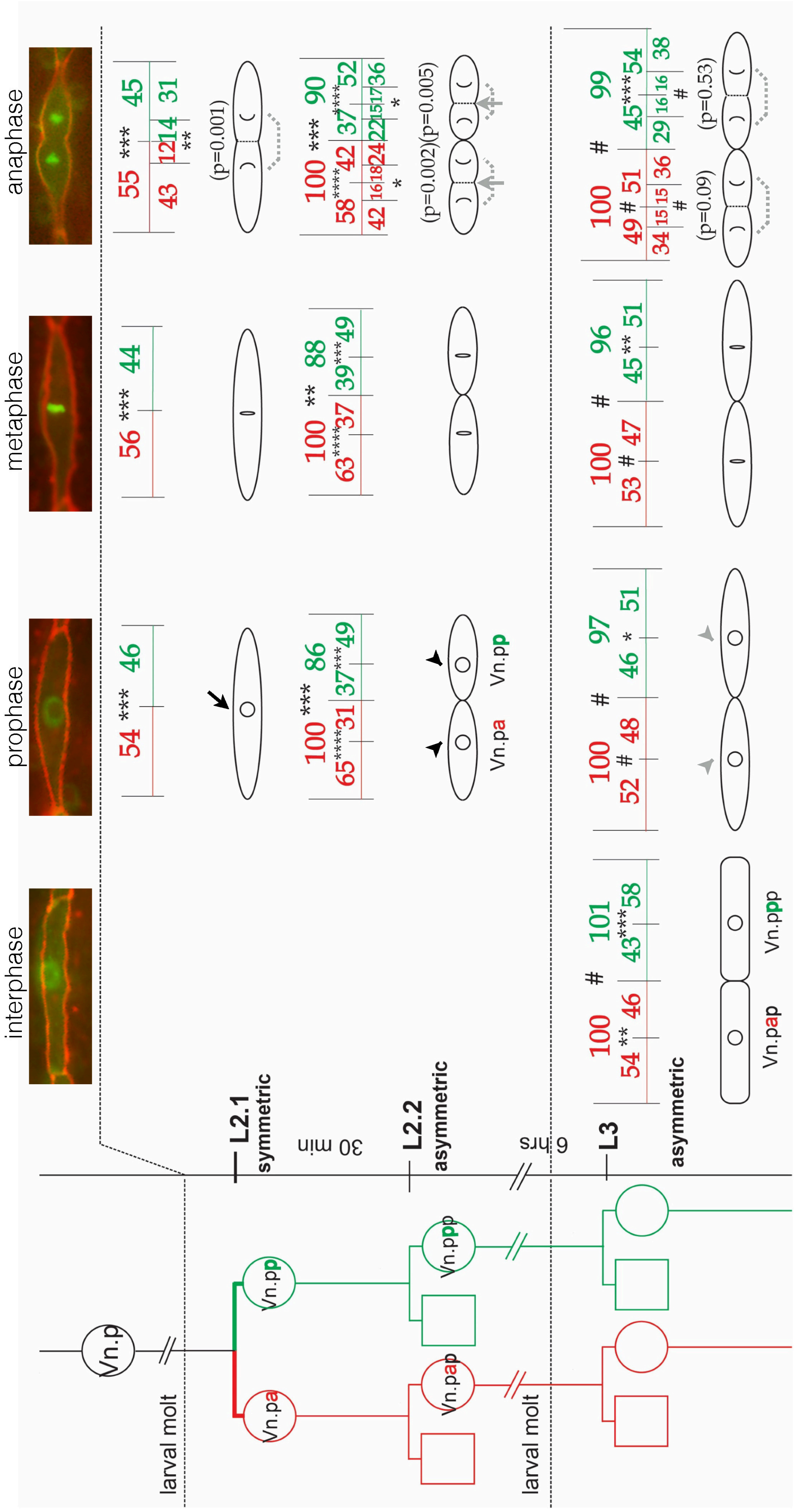
interphase
prophase
metaphase
anaphase
L2.1
symmetric
30 min
L2.2
asymmetric
6 hrs
L3
asymmetric
larval molt
larval molt
Vn.p
Vn.pa
Vn.pp
Vn.pap
Vn.ppp
(p=0.001)
(p=0.002)
(p=0.005)
(p=0.09)
(p=0.53)

Supplementary Figure S5. Chromosomal positioning during V-lineage seam cell L2.1 symmetric, L2.2 asymmetric and L3 asymmetric divisions after *nmy-2* knockdown. Strain AW1116 was bleach synchronised to L1 and transferred to *nmy-2* RNAi plates at 25°C. Measurements were taken in the same way as in Supplementary Fig. S3. All four wild-type seam cell division characteristics, namely the off-centre nuclear (black arrow) and spindle (gray dashed brackets) placement, the pairing of the anterior and posterior-branch nuclei (black arrowheads), and the displacement of the cleavage furrow at L2.2 asymmetric division (gray arrows), were recapitulated in this *nmy-2* knockdown dataset, suggesting that *nmy-2* is perhaps not involved in generating or maintaining physical division asymmetry during seam cell divisions.

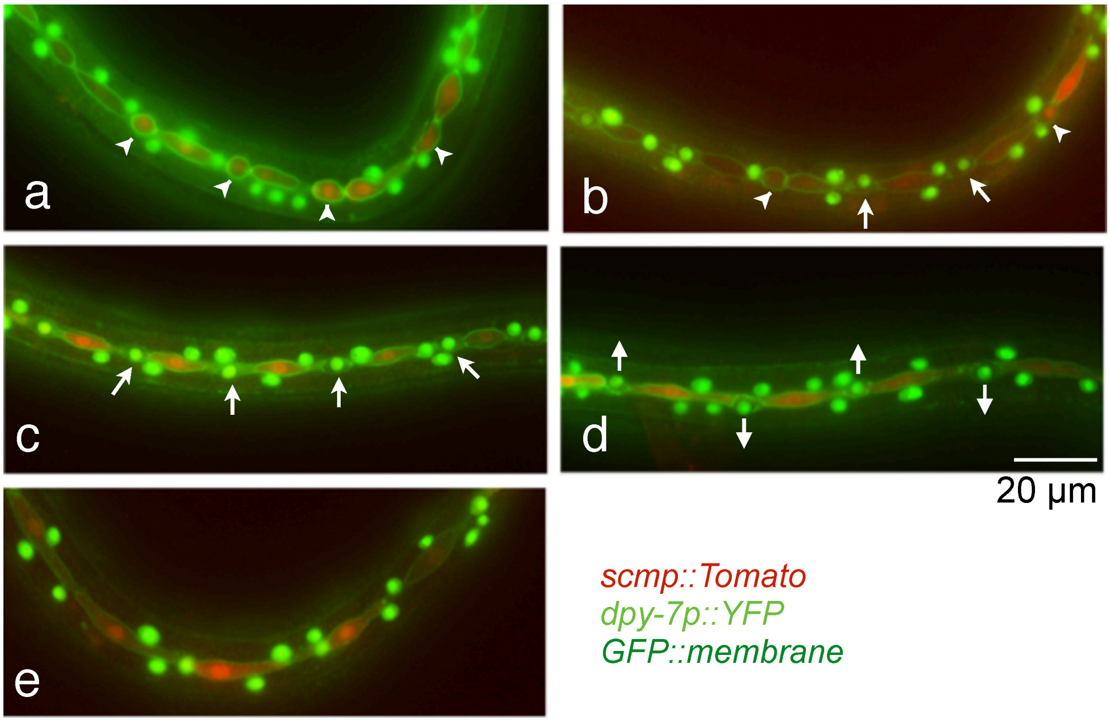

Supplementary Figure S6. The dual-colour fate reporter strain illustrates the sequence of events during hypodermal fate acquisition. Following the L3 division, anterior daughter differentiation was captured by a colour change using the strain (AW1015). **a**) The anterior daughters initially expressed the red seam marker after division (arrowheads), even as the two daughters on the left rounded up. **b**) Next, while some of the rounded anterior daughters continued to express the red seam marker (arrowheads), others started expressing the green hypodermal marker (arrows). **c**) All anterior daughters now expressed the differentiative marker (arrows), before **d**) some moved dorsally and some ventrally out of the seam line. **e**) Eventually, all the anterior daughters fused with the hyp7 syncytium and the posterior seam daughters elongated to reconstitute the seam line. Individual panels are snapshots of different worms, but the sequence of events shown here is typical of anterior differentiation.

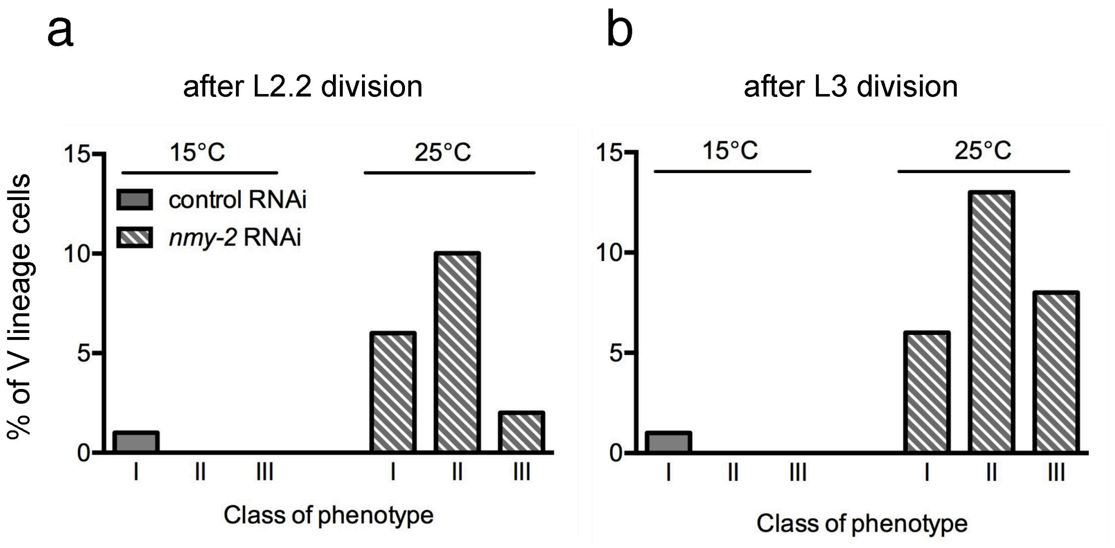

Supplementary Figure S7. Quantification of cell fate mis-specification phenotypes following *nmy-2* knockdown. Quantification data from Fig. 3e-f are represented here as a percentage of total V lineage cells after the L2.2 and L3 asymmetric divisions, by dividing the total number of V cells exhibiting each phenotype by the total number of V cells present.

	15°C	17°C	20°C	25°C
WT	✔	✔	✔	✔
wrm-1	✔	✔	✔	✕
nmy-2	✔	✔	✔	✕
nmy-2; wrm-1	✔	✔	✕	✕

Supplementary Figure S8. *nmy-2* inactivation enhances the phenotype of WNT/β-catenin asymmetry pathway component knockdown during embryonic development**.** A single L4 animal of wild-type, *nmy-2* and *wrm-1* single mutants, and the *nmy-2;wrm-1* double mutant (strains AW335, EW95, AW785 and AW861, respectively) was placed at the indicated temperatures and its embryonic viability was assessed. ✔ denotes that the animal produced viable progeny and ✕ denotes that no viable progeny was produced. n=15 for each sample. Three independent replicates of the experiment were performed.

Supplementary Methods

Chromosomal positioning analysis

Worms of the desired stage were mounted in 0.5% phenoxypropanol on 2% agarose pads. The DNA marker in *heIs63[wrt- 2p:GFP::PH;wrt-2p::GFP::H2B;lin-48p::mCherry]* and the apical junction marker in *ouIs21[ajm-1p::mCherry]* were used to measure chromosomal positioning with respect to the ends of the cells and to the ingressing cleavage furrow as follows. Two sets of images for each pertinent cell were taken on the GFP and Rhodamine channels on the Zeiss Axiophot fluorescent microscope, one set with *wrt-2p::GFP::H2B* in focus and the other set with *ajm-1p::mCherry* in focus. Post-acquisition analysis involves using ImageJ to overlay the GFP channel focusing on *wrt-2p::GFP::H2B* with the Rhodamine channel focusing on *ajm-1p::mCherry*. This was done because of the tapered shape of the seam cells in three-dimensions. Cell size as outlined by the PH membrane marker would vary depending on the Z plane, so instead *ajm-1p::mCherry* on the apical surface was used to show the “true” cellular boundary. After cell identity and orientation were manually assessed, distance measurements were made in ImageJ. V1-4 lineages were pooled for the analysis because they share the same division patterns. V5 has a different lineage and V6, while sharing the V1-4 lineage, was obscured by the *lin-48p::mCherry* co-injection marker in *heIs63* so they were excluded from the analysis. For interphase through metaphase, distances between anterior and posterior ends of the cell, and between ends of the cell and the DNA were measured. For late anaphase, additional distances between the two sets of daughter chromosomes and

between the chromosome and the cleavage furrow were measured. All measurements within a cell were normalised to the full A-P length of that cell, which was assigned a value of 100. Two-tailed *t*-tests were performed to determine statistical significance, using paired *t*-test for anterior versus posterior measurements within the same cell and unpaired *t*-test assuming unequal variances for measurements comparing different cells belonging to the anterior versus posterior lineage branches.
